# Supervised Physical Therapy and Polymyositis/Dermatomyositis—A Systematic Review of the Literature

**DOI:** 10.3390/neurolint12030015

**Published:** 2020-11-24

**Authors:** Bruno Corrado, Gianluca Ciardi, Laura Lucignano

**Affiliations:** Department of Public Health, Federico II University of Naples, Via S. Pansini 5, 80131 Naples, Italy; gianluca.ciardi@unina.it (G.C.); laura.lucignano95@gmail.com (L.L.)

**Keywords:** polymyositis, dermatomyositis, physical therapy, rehabilitation, quality of life

## Abstract

Objective: to find the most up-to-date evidence of the effectiveness and safety of supervised physical therapy in polymyositis/dermatomyositis patients. Methods: a systematic review of the literature in the main scientific databases was carried out. We searched for randomized controlled trials concerning supervised physical therapy and polymyositis/dermatomyositis. The PICOS method was used for the formulation of the clinical query. Methodological quality and the level of evidence of the included studies were assessed using the modified Jadad scale and the Oxford Centre for Evidence-Based Medicine Levels of Evidence guide, respectively. Results: a total of 2591 articles were found. By applying the inclusion/exclusion criteria, six randomized controlled clinical trials were admitted to the final phase of the review. The compared approaches concerned supervised exercise programs based on strategies of muscle strengthening or aerobic work. Following these exercises, an increase in the maximum rate of oxygen consumption, a decrease in creatine phosphokinase levels, an enhancement in the patient’s aerobic performance and an improvement in the quality of life indexes were registered. The methodological quality of the included studies ranged from 3 to 4.5. All the studies were classified as presenting an evidence level of 2b. Conclusions: supervised physical therapy in polymyositis/dermatomyositis is an effective, safe and free-of-contraindications tool to be used both in the acute and in the established phases of the pathology. However, further and higher-quality studies are necessary to confirm those findings, to clarify the timing of exercise delivery and to guide the choice towards different types of muscle contraction exercises.

## 1. Introduction

The terms polymyositis (PM) and dermatomyositis (DM) identify the two most common syndromes of the group of idiopathic inflammatory myopathies (IIM). Sporadic inclusion body myositis (sIBM) and immuno-mediated necrotising myopathy (IMNM) complete the group [1,2,3,4]. The common feature of IIM is the presence of inflammatory exudate and autoantibodies within the muscles. IIM are thought to arise from a combination of genetic and environmental factors.

Variations in several genes have been identified, which may increase a person’s risk of developing IIM [5]. Exposure to certain environmental factors may then trigger the disorder: UV light, drugs, infections or an unhealthy lifestyle [6]. In addition, in recent years there has been a growing interest in the paraneoplastic forms of IIM, which are quite frequent [6]. Different organs and systems can be compromised in IIM: heart, lung, skin, gastrointestinal tract, and skeleton [7,8,9,10,11].

Epidemiological studies conducted on the North American population showed an annual incidence of IIM of about 4–7/100,000 and a prevalence between 10 and 30/100,000 [12].

The diagnostic criteria of IIM are still under discussion, and often these pathologies are misunderstood [3,6]. Only cases characterised by skin involvement (DM) can be identified according to the proposal of Bohan and Peter: (a) symmetrical weakness of the limb girdle muscles and anterior neck flexors, progressing over weeks to months, (b) elevation in serum of the skeletal muscle enzymes creatine kinase (CK), aldolase (ALD), glutamate oxaloacetate transaminase (GOT), glutamate pyruvic transaminase (GPT), lactate dehydrogenase (LDH), (c) electromyographic triad of small, short, polyphasic motor units, fibrillations, positive sharp waves (PSWs) and insertional irritability and bizarre, high-frequency repetitive discharges, (d) muscle biopsy evidence of necrosis of myofibers, phagocytosis, regeneration with basophils, large vesicular sarcolemmal nuclei and prominent nucleoli, atrophy in a perifascicular distribution, variation in fibre size and inflammatory exudates and (e) any one of the characteristic dermatologic features of the rash of DM [13].

For diagnosis, the presence of a rash and of at least one/two muscular signs is necessary [13]. Traditionally, all forms that do not meet the stated criteria are classified as PM, and only the presence of autoantibodies allows a better differentiation [6]. Myositis-specific antibodies (MSA) have a specificity of 90% and are associated with different phenotypes; within this group, antisynthetase antibodies are among the most studied (anti-Jo-1, anti-Mi-2, anti-MDA5, anti-TIF, anti-SAE, anti-NXP) [14,15].

From a clinical point of view, the onset of PM and DM is usually subacute, with the progressive appearance of symmetrical and proximal asthenia of the limbs and trunk; the patient typically cannot lift his arms, comb his hair, climb the stairs or get up from the ground after a fall [16]. The sensory nervous system and tendon reflexes are generally not involved, whereas in the patients with more aggressive onset, the weakness of the diaphragm makes it necessary to have ventilatory support [17]. Other target systems of DM and PM are the swallowing apparatus—with the onset of dysphagia and, in the most serious cases, of pneumonia ab ingestis—and the circulatory system—with the onset of arrhythmias, myocarditis and endocarditis in the chronic phase of DM [9,10,11].

Therapy for DM and PM usually begins with high-dose oral prednisone (1 mg/kg/day), which should be subsequently tapered off slowly based on patients’ clinical response [18]. Intravenous immunoglobulins are a viable treatment option as second-line therapy [19,20]. Association of prednisone with intravenous immunoglobulins may be considered in patients with systemic complications ab initio [21]. In patients who failed to respond to high-dose prednisone, the first-line therapy includes metothrexate or aziathioprine. In case of failure of all the above-mentioned treatments, the main options are combined therapy of methotrexate and azathioprine, mycophenolate mofetil or monoclonal antibodies (rituximab and sifalinumab) [18,22,23,24,25,26,27].

Physical therapy has progressively acquired a central role in the management of neuromuscular disorders [28,29,30,31]. However, in the recent past, scientific knowledge about exercising in patients with muscle diseases was limited, and for this reason, physicians had often trouble prescribing it. Nowadays, it is well known that strength training or aerobic exercise programs might optimise muscle and cardiorespiratory functions and prevent additional disuse atrophy and deconditioning in people with a muscle disease [32].

In supervised physical therapy (PT), patients spend more than 50% of the exercise time on training alone, following the advice of a physical therapist. During the remaining 50% of the exercise time, patients exercise under the control of a physiotherapist who oversees the development of the exercise program. Supervised PT has proved to be as effective as non-supervised PT for some cardiovascular diseases, like intermittent claudication [33].

The purposes of this systematic review were (a) to verify the safety and efficacy of supervised PT in people with PM/DM and (b) to provide a clear indication of the different types of exercises in the management of PM/DM.

## 2. Materials and Methods

The PICOS method was used to formulate the clinical query:

– P (population): patients with PM/DM;
– I (intervention): physical exercise/supervised/physiotherapy/physical therapy;
– C (comparison): inactive control groups;
– O (outcomes): improvements in muscular strenght/quality of life;
– S (study type): randomized controlled trials (RCTs).

This systematic review followed the Preferred Reporting Reviews and Meta-Analysis (PRISMA) reporting guidelines for systematic review or meta-analysis of intervention trials [34].

### 2.1. Study Eligibility Criteria and Report Eligibility Criteria

Studies were eligible for inclusion in this systematic review if (1) they were in the English language; (2) they had been published any time before May 2019; (3) they enrolled patients aged > 18 years with PD/DM; (4) supervised PT was applied to at least a proportion of the patients; and (5) appropriate data were provided for the evaluation of the results.

Studies were excluded from this review if other than RCTs. Textbook extracts, conference proceedings, expert opinion, consensus panels were excluded.

### 2.2. Search Strategy

To identify relevant studies, two investigators independently searched the PubMed, Embase, Web of Science and Cochrane Library databases using the search terms listed in Table 1.

### 2.3. Study Selection

The two investigators first scanned the study titles and abstracts and discarded obviously ineligible articles. The full texts of the remaining studies were then reviewed to check that all inclusion criteria were met. Disagreements between reviewers were resolved by consensus.

### 2.4. Data Collection Process

Data were extracted from the included studies using a data extraction form (Table 2). Data were extracted by one investigator and crosschecked by the other. The following data were recorded: general information concerning the study (lead author and year of publication), study design, number of participants, interventions, outcome measures, methodological quality score, and level of evidence. Disagreements between the two investigators were resolved by discussion and, if necessary, by consultation with a third investigator.

### 2.5. Methodological Quality and Level of Evidence Assessment

Methodological quality and level of evidence were assessed independently by two of the investigators. Methodological quality was assessed using the modified Jadad scale [41]. The level of evidence was assessed using the Oxford Centre for Evidence-Based Medicine (OCEBM) Levels of Evidence guide [42]. Publication bias was not assessed because of the very small number of selected studies.

## 3. Results

**Figure 1:**
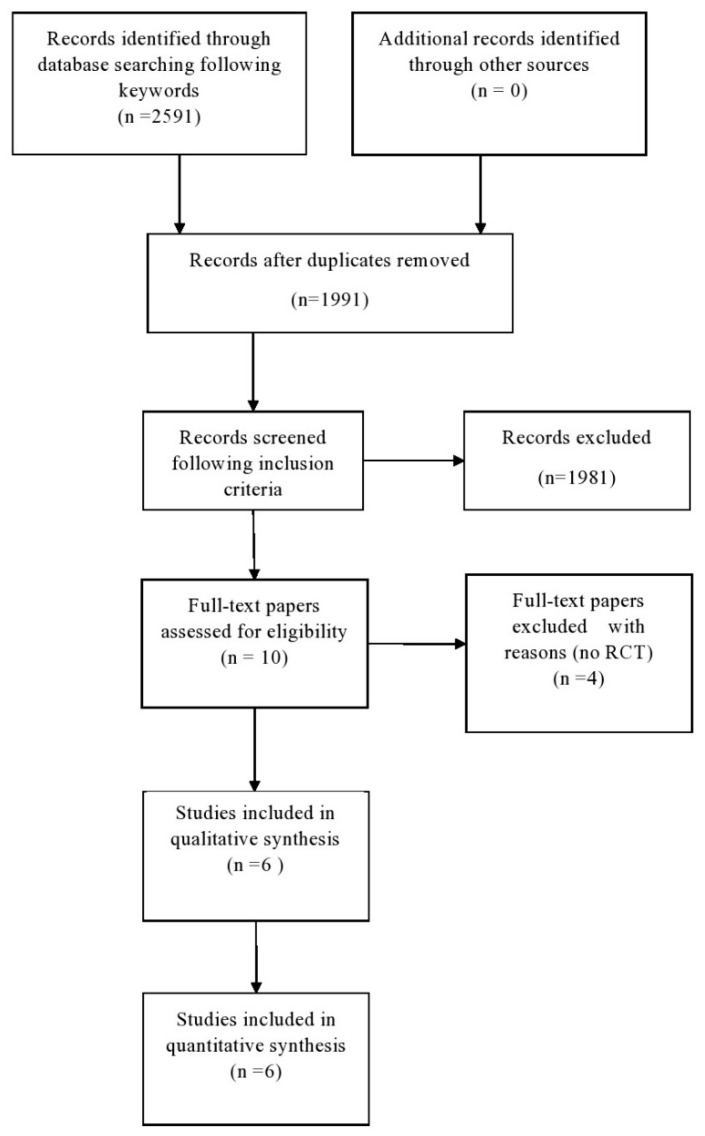
Selection of articles for the review.

The database search yielded 2591 results. Figure 1 shows the process of gradual sorting of the items found. With an initial screening, all the duplicates were eliminated, thus excluding 600 articles. A second screening of the evidence by title and abstract was performed in order to exclude articles not related to supervised PT and PM/DM and types of publication other than those stated in the inclusion criteria. The second screening allowed the elimination of a further 1981 results; a total of 10 articles were therefore evaluated in the full-text version. These were divided as follows: six RCTs and four studies on a single cohort. The six studies of the first group were therefore admitted to the final phase of the review.

The objective of the clinical trial carried out by Wiesinger et al. was to evaluate the benefit of a six-week aerobic training, in association with drug treatment, in patients with stable PM/DM [39]. Forteen patients were recruited and divided into an exercise group (EG, *n* = 7) and a control group (CG, *n* = 7). Baseline and endpoint measurements included: the Functional Assessment Screening Questionnaire for activities of daily living (ADL), the Visual Analogue Scale (VAS), the peak isometric torque (PIT) generated by hip flexors and knee extensors muscles, maximum oxygen consumption (VO_2_ max) and creatine phosphokinase (CPK) serum levels. The EG took part in a six-week training program that included bicycle exercise and step aerobics. The CG did not participate in any training. At the end of the study, the authors reported a significant improvement in ADL performance, muscle strength and VO_2_ max in the EG. The modified Jadad scale score for this RCT was 4/8. The study was classified as having an evidence level of 2b according to the OCEBM-Levels of Evidence guide.

Wiesinger et al. conducted a second RCT to evaluate the benefit of a long-term (six months) physical training in patients with stable PM/DM [40]. Thirteen patients were included in this study and were divided into an EG (*n* = 8) and a CG (*n* = 5). The physical training program and the outcomes were the same as in the previous trial [39]. The endpoint was set at six months. The comparison between endpoint and baseline values in EC and CG patients confirmed the trend of the previous study, with a 28% increase in VO_2_ max, improved muscle strength and better ADL performance in treated patients. The authors concluded that long-term physical training is recommended as part of a comprehensive rehabilitation management in clinically stable DM/PM patients. The modified Jadad scale score for this RCT was 4/8. The study was classified as having an evidence level of 2b according to the OCEBM-Levels of Evidence guide.

Munters et al. carried out two different studies about physical exercise in patients with PM/DM. During the first RCT, a 12-week endurance training program was proposed in addition to immunosuppressive drug therapy [35]. The authors selected 15 patients with stable PM/DM and divided them into an EG (*n* = 9) and a CG (*n* = 6). The EG performed an endurance training program consisting of warm-up cycling followed by more intense cycling, muscular endurance exercise, engaging both the upper and the lower limbs, and muscle stretch. The CG were advised not to change their physical activity level during the 12 weeks. The assessed outcomes were: VO_2_ max and the associated power output during a progressive cycling test (65% of VO_2_ max), lactate levels in the vastus lateralis muscle measured with microdialysis, citrate synthase (CS) and β-hydroxyacyl-CoA dehydrogenase (β-HAD) activities in muscle biopsies and clinical improvement according to the criteria of the International Myositis Assessment and Clinical Studies Group (IMACS). At the end of the trial, EG patients showed increased aerobic capacity, decreased lactate levels at exhaustion, increased CS and β-HAD activities. The authors stated that endurance exercise is effective, in addition to immunosuppressive treatment, in the management of patients with stable PM/DM. The modified Jadad scale score for this RCT was 3.5/8. The study was classified as providing an evidence level of 2b according to the OCEBM-Levels of Evidence guide.

Afterwards, Munters et al. carried out a multicentre RCT on the effects of a 12-week endurance exercise program on health, disability, VO_2_ max and disease activity in patients with established PM and DM [36]. Twenty-one patients were selected and divided into two groups: an EG (*n* = 11) and a CG (*n* = 10). The following outcomes were assessed: health (SF-36), muscle performance (five voluntary repetition maximum—5 VRM), ADL, patient preference (McMaster Toronto Arthritis Patience Preference Disability Questionnaire), VO_2_ max, and disease activity (IMACS criteria). The EG carried out a 12-week exercise program consisting of about 30 min of 50–70% VO_2_ max exercise bike and 20 min of endurance exercises for knee extensors muscles at 30–40% of the VRM. Individuals in the CG were advised not to change their physical activity level. All patients were given a daily exercise diary and were supervised by the same physical therapist at their respective centre. At the end of the treatement period, the EG patients showed improved quality of life (for both physical and mental health domains of the Short Form-36 (SF36) questionnaire), better VO_2_ max, improved muscular performance and reduced disease activity. The modified Jadad scale score for this RCT was 4.5/8. The study was classified as having an evidence level of 2b according to the OCEBM-Levels of Evidence guide.

The Karolinska Institute research group carried out a third RCT led by Alexanderson et al. [37]. In this trial, 19 patients with recent-onset PM/DM were randomised into two groups: an EG (*n* = 10) and a CG (*n* = 9). The EG carried out a 12-week, 5 days/week resistive home exercise with telephone support and encouragement, followed by additional 12 weeks of twice-a-week home or gym exercise. The CG performed a 24-week, 5 days/week range-of-motion exercise. Patients in the CG group without inflammatory infiltrates in muscle biopsies at 24 weeks were invited to the 12-week resistive home exercises. All patients were included in the RCT after introduction of high-dose prednisolone. The following assessment tools were used: CPK, Myositis Functional Index (MFI), Nottingham Health Profile (NHP), 8-min submaximal treadmill test and the Borg Rate of Perceived Exertion (RPE) scale. At 24 and 52 weeks, both groups improved in muscle performance and aerobic capacity. At 104 weeks, only the EG maintained those positive results. No further significant effects were reported. The modified Jadad scale score for this RCT was 4.5/8. The study was classified as providing an evidence level of 2b according to the OCEBM-Levels of Evidence guide.

Tiffreau et al. evaluated the functional impact and effect on quality of life at 12 months of a 4-week standardised exercise program in patients with active PM [38]. Twenty-one PM patients were included in the study, divided into an EG (*n* = 10) and a CG (*n* = 11), both undergoing steroid treatment. The Health Assessment Questionnaire Disability Index (HAQ-DI), the SF-36 questionnaire, the Kendall Manual Muscle Test (MMT), the Motor Function Measure (MFM), the VAS, the 6-Minute Walking Test (6MWT), and the serum levels of C-reactive protein (CRP) and CPK were evaluated. The EG underwent a four-week standardized, hospital-based rehabilitation program, followed by a personalized, self-managed, home-based program. The rehabilitation program was focused on muscle strength training, chest expansion, increased joint range of motion, better gait and transfers, and improved aerobic capacity. The CG remained inactive. At the final follow-up (12 months), the EG showed a lower degree of impairment (HAQ-DI) and better scores for quality of life and pain levels compared to the CG. Therefore, the combination of a four-week standardized rehabilitation program and a personalized, home-based rehabilitation program was found to be safe and effective in patients with active PM. The modified Jadad scale score for this RCT was 3/8. The study was classified as having an evidence level of 2b according to the OCEBM-Levels of Evidence guide.

## 4. Discussion

It is well known that physical activity positively impacts health-related quality of life and well-being. However, for a long while it has been wrongly believed that physical exercise could be harmful for patients with miopathies and, in particular, for patients with inflammatory miopathies. The major concern for healthcare providers was that exercise might raise muscle inflammation in patients with PM/DM, with a resulting worsening of muscle weakness. By contrast, the first finding that appears to be common to all the reviewed studies is that supervised PT represents a safe tool in the management of patients with PM/DM, both active or established [35,36,37,38,39,40]. Indeed, none of the selected studies showed an increased inflammation risk in patients with PM/DM who underwent a supervised exercise program. In addition, physical activity has proven to have an antiinflammatory effect by increasing the levels of peripheral blood antiinflammatory cytokines and by reducing those of inflammatory ones. The reviewed studies also stated that supervised PT has an adjuvant effect on immunosuppressive drug therapy. This appears to confirm what has been described in several non-randomised clinical trials. For example, the 2000 case series by Alexanderson et al. stated that physical exercise did not raise inflammation in patients with active PM/DM and therefore it should be always combined with drug therapy [43].

Both aerobic exercise and resistance training are used in the management of patients affected by PD/DM. In the selected trials, aerobic exercise proved to enhance the performance of patients with PM/DM, both by the increase in VO_2_ max values and by the improvement of mitochondrial activity and oxygen uptake of skeletal muscles. It has been demonstrated that an aerobic training program resulted in a 28% increase of VO_2_ max after 6 months and in an increment of CS and β-HAD activities after 12 weeks. With regard to the type of muscle contraction to be recommended to patients with PM/DM during aerobic performance, the reviewed studies showed that concentric contractions were more advisable. As a matter of fact, during eccentric contractions, muscle fibers are stretched under tension by a force greater than that generated by the muscle and therefore they could be damaged. It follows that sport activities that could be performed without risk in patients with PM/DM are cycling, walking and jogging on a level ground.

The selected studies stated that also resistance training could be suggested to patients with PM/DM, but with some limitations. Resistance training is any exercise that causes the muscles to contract against an external resistance with the expectation of increases in strength, tone, mass and/or endurance. Resistance training is classified according to its intensity. High-intensity resistance training was not recommended for patients with PM/DM because it causes microscopic damage or tears to the muscle fibers. The combination of low-intensity resistance trianing (e.g., 20–30%, one repetition maximum [1RM]) with blood flow restriction (LRT–BFR) has been alleged to induce similar gains in muscle mass and strength compared with conventional high-intensity resistance, without causing damage to the muscles.

The ideal duration of the minimum supervised PT should be further investigated. In this review, it emerged that supervised exercise programs that are limited to the short term (4, 6 or 12 weeks) as well as decidedly more durable approaches (24 weeks and up to 6 months) have all achieved statistically significant results in contrasting the pathology and improving patient performance.

The effect of supervised PT on patients’ quality of life is also important. Alemo Munters et al. documented a statistically significant improvement in the SF-36 scores in the exercise group, as did Tiffreau [36,38]. This finding is also confirmed in studies other than RCT: in a single cohort study, Mattar et al. verified a statistically significant improvement in the quality of life of patients with PM/DM subjected to physical exercise and partial restriction of blood flow [44]. Additional statistically significant effects of supervised PT documented by individual authors concern the activity of mitochondrial enzymes, muscle strength (assessed by isokinetic exercise) and pain levels experienced: these effects can all be traced back to the general benefits of physical exercise, especially resistive, and find further literary evidence in previous cohort studies [35,37,39,44,45].

According to the modified Jadad scale, the selected studies’ methodological quality ranged from 3 to 4.5. The main criticisms concerned the method of randomisation, which was often not well described or almost inappropriate, and the lack of double blinding. The OCEBM Levels of Evidence guide allowed to attribute to all the included studies a score of 2b (low quality RCTs). In conclusion, this systematic review had a moderate strenght of evidence.

The main limitations of this systematic review are the small number of included studies and the resultant inability to explore publication bias.

In conclusion, supervised PT has been found to be a safe and effective tool in the management of patients with PM/DM, both active or established, with no contraindications or known adverse effects. The present systematic review made it possible to achieve moderate strength of evidence for the following benefits of an approach based on supervised aerobic/resistance training: (a) decreased muscle inflammatory indices, (b) increased aerobic capacity of the patient and, consequently, autonomy in ADL, (c) improved quality-of-life indexes and (d) improvement of muscle strength. On the other hand, sufficient evidence has not been obtained about some aspects of the exercise approach, for example, the timing of session delivery and the most effective type of muscle contraction. New future trials, therefore, will have to clarify these aspects of physical exercise in patients with PM/DM.

## Figures and Tables

**Table 1:** Keywords used for the search.

Keyword 1	Keyword 2	Boolean Operator
Dermatomyositis	Physical exercise	AND/OR
	Physical activity	
	Physical therapy	
	Physiotherapy	
Polymyiositis/Dermatomyositis	Physical exercise	AND/OR
	Supervised	
	Physical therapy	
	Physiotherapy	
Autoimmune inflammatory Myopathies	Physical exercise	AND/OR
	Supervised	
	Physical therapy	
	Physiotherapy	
Idiopathic Inflammatory Myopathies	Physical exercise	AND/OR
	Supervised	
	Physical therapy	
	Physiotherapy	

**Table 2:** Characteristics of the included studies. EG, exercise group; CG, control group; wk, week.

Study	Design	Participants	Intervention	Outcome Measures	Modified Jadad Scale	Level of Evidence
Alemo Munters et al. (2013 [35])	RCT	*n* = 15	EG = cyclette, strenght exercise, stretching 3/7 × 12 wk CG = usual physical activity	VO2 Max Citrate synthase (CS) activity in muscle biopsy Bhydroxyacyl-CoA dehydrogenase (b-HAD) activity in muscle biopsy Myositis Assessment and Clinical Studies Group (IMACS) Lactate levels Follow-up: 0, 12 wk	3.5/8	2b
Alemo Munters et al. (2013 [36])	RCT	*n* = 23	EG = cycling, strength exercise 3/7 × 12 wk CG = usual physical activity	VO2 Max SF-36 Activities of daily living (ADL) McMaster Toronto Arthritis Patient Preference Disability Questionnaire International Myositis Assessment and Clinical Studies criteria ofimprovement of the 6-item core set Follow-up = 0, 12, 52 wk	4.5/8	2b
Alexanderson et al. (2014 [37])	RCT	*n* = 19	EG = resistive home exercise program and brisk walking; home/gym exercise during the follow up 7/7 × 12 wk (each leg) CG = 15 min range of motion (ROM) exercise program 5 days a week	Creatine phosphokinase (CPK) FI (Myositis Functional Index) Nottingham Health Profile (NHP) 8 min submaximal treadmill test Borg Rate of Perceived Exertion (RPE) scale. Follow-up = 0, 52, 78, 104 wk	4.5/8	2b
Tiffreau et al. (2016 [38])	RCT	*n* = 21	EG = strength exercise, breathing exercise, walking, aerobic exercise, postural changes 5/7 × 4 wk CG = no intervention	Questionnaire Disability Index (HAQ-DI) SF-36 Kendall Manual Muscle Test Motor Function Measure VAS 6-Minute Walking Test Serum level of C-reactive protein (CRP)/CPK Follow-up: 0, 4, 12 wk	3/8	2b
Wiesinger et al. (1998 [39])	RCT	*n* = 14	EG = cyclette + step with progressive resistance 2–3/7 × 6 wk CG = no intervention	ADL score Peak isometric torque (PIT) Peak oxygen consumption (VO2max) CPK levels Follow-up = 0, 6 wk	4/8	2b
Wiesinger et al. (1998 [40])	RCT	*n* = 13	EG = cyclette + step with progressive resistance 3/7 × 24 wk CG = no intervention	CPK levels ADL score Peak isometric torque (PIT) VAS VO2Max Follow-up: 0, 24 wk	4/8	2b
